# An assessment of genome annotation coverage across the bacterial tree of life

**DOI:** 10.1099/mgen.0.000341

**Published:** 2020-03-03

**Authors:** Briallen Lobb, Benjamin Jean-Marie Tremblay, Gabriel Moreno-Hagelsieb, Andrew C. Doxey

**Affiliations:** ^1^​ Department of Biology, University of Waterloo, 200 University Avenue West, Waterloo, ON N2L 3G1, Canada; ^2^​ Department of Biology, Wilfrid Laurier University, 75 University Avenue West, Waterloo, ON, Canada

**Keywords:** genome annotation, functional annotation, gene function prediction, bacterial genomics, phylogenomics, tree of life

## Abstract

Although gene-finding in bacterial genomes is relatively straightforward, the automated assignment of gene function is still challenging, resulting in a vast quantity of hypothetical sequences of unknown function. But how prevalent are hypothetical sequences across bacteria, what proportion of genes in different bacterial genomes remain unannotated, and what factors affect annotation completeness? To address these questions, we surveyed over 27 000 bacterial genomes from the Genome Taxonomy Database, and measured genome annotation completeness as a function of annotation method, taxonomy, genome size, 'research bias' and publication date. Our analysis revealed that 52 and 79 % of the average bacterial proteome could be functionally annotated based on protein and domain-based homology searches, respectively. Annotation coverage using protein homology search varied significantly from as low as 14 % in some species to as high as 98 % in others. We found that taxonomy is a major factor influencing annotation completeness, with distinct trends observed across the microbial tree (e.g. the lowest level of completeness was found in the *Patescibacteria* lineage). Most lineages showed a significant association between genome size and annotation incompleteness, likely reflecting a greater degree of uncharacterized sequences in 'accessory' proteomes than in 'core' proteomes. Finally, research bias, as measured by publication volume, was also an important factor influencing genome annotation completeness, with early model organisms showing high completeness levels relative to other genomes in their own taxonomic lineages. Our work highlights the disparity in annotation coverage across the bacterial tree of life and emphasizes a need for more experimental characterization of accessory proteomes as well as understudied lineages.

## Data Summary

Bacterial genomes from AnnoTree [1] and their Pfam and KEGG (Kyoto Encyclopedia of Genes and Genomes) annotations (gtdb_r86_bac_genomic_files.tar.gz, gtdb_r86_bac_pfam_tophits.tar.gz and gtdb_r86_bac_ko_tophits.tar.gz, respectively) were retrieved from https://data.ace.uq.edu.au/public/misc_downloads/annotree/r86/. Metadata from the Genome Taxonomy Database (GTDB) [2] were retrieved from https://data.ace.uq.edu.au/public/gtdb/data/releases/release86/86.1/bac120_metadata_r86.1.tsv. A data table listing frequencies of annotated versus unannotated gene counts can also be found at: https://github.com/doxeylab/genomeAnnotationCoverage.

**Impact Statement**

To what extent can bacterial genomes be assigned function is an important question in automated genome annotation. To investigate this question, we annotated over 27 000 bacterial genomes from the Genome Taxonomy Database using common bioinformatic methods, and evaluated the influence of different factors on annotation completeness. Annotation coverage, defined as the percentage of predicted protein sequences that could be assigned functions, ranged from 14 to 98%, with a mean of ~52 %. Mean annotation coverage increased to 79 % when using domain-based methods. Additional significant factors related to annotation coverage include taxa, genome size and 'research bias' (i.e. the increased annotation coverage in genomes of model organisms). Our work also highlighted the *Patescibacteria* lineage as a group associated with the lowest degree of annotation coverage, potentially reflecting a unique gene content and biology to be found in these organisms.

## Introduction

Genome annotation relies primarily on the detection of homology between newly identified genes/proteins and previously annotated sequences. As a general summary of this process, genes predicted in newly sequenced genomes or metagenomes are translated and compared against reference databases to identify homologues, with functional annotations being transferred from those homologues to the query proteins [3]. Although complicated by varying definitions of ‘function’ and ‘annotation’, homology-based annotation transfer has been systematically explored, revealing reasonable success rates (upwards of 60–70 % accuracy) based on assessment of Gene Ontology (GO) term prediction [4, 5]. Studies of early model organisms, such as *Escherichia coli *, *Bacillus subtilis* and *Caulobacter crescentus *, are a major source of experimentally derived functional annotations. Therefore, it is important to note that such limited sources can be expected to result in biases in genome annotation, with a greater success rate in species that are phylogenetically closer to these and other commonly studies species [6].

In the digital and post-genomic age, functional annotations can be transferred between sequences faster and more broadly than ever before, through a variety of computational methods and pipelines. Standard approaches include sequence-to-sequence searches such as blast or sequence-to-model searches (e.g. HMMscan) that scan newly identified sequences against models of protein and/or domain families [7]. Profile-based methods that use position-specific scoring matrices (PSSMs) or hidden Markov models (HMMs) such as Pfam and the National Center for Biotechnology Information's (NCBI’s) Conserved Domain Database [8–10] are among the most sensitive approaches for protein classification, as these are capable of detecting distant matches to protein and/or protein domain families. Domain families are used to find matches to building blocks of proteins, such as enzymatic or binding domains, sometimes allowing functional information transfer even in the absence of a full protein match [9].

Both sequence-to-sequence and profile-based methods are implemented in common annotation pipelines such as Prokka [11], the Joint Genome Institute Microbial Annotation Pipeline [12] and NCBI’s Prokaryotic Genome Annotation Pipeline [13]. Annotation pipelines may also integrate a variety of methods and databases, and/or allow users to customize options towards specific reference databases or taxonomic lineages. Commonly used reference databases include UniProt/SwissProt, as well as the NCBI’s reference sequence (RefSeq) database, and its non-redundant protein database. Other reference databases of protein and/or domain families include TIGRFAMs [8], FIGfams [14], COG [15] and Pfam [9].

Even with sequence databases growing at an exponential rate and with ongoing expansion of annotation information in reference databases, well-studied organisms still have significant proportions of their coding sequences (CDSs) functionally unannotated [7, 16–18]. When predicted protein sequences cannot be functionally annotated, they are typically classified as ‘hypothetical’ proteins, or sometimes as ‘conserved hypothetical’ proteins if they are commonly detected in the genomes of numerous organisms [19, 20]. These hypothetical sequences consist of proteins of unknown function as well as potential pseudogenes and even spurious gene predictions [18, 21].

An important question in genome-wide functional annotation is to what degree a genome (or more specifically, a proteome) can be assigned function [22, 23]. Interestingly, across different bacterial species/genomes there is considerable variation in the completeness of genome annotations reported in the literature and in databases [6, 24]. For example, according to the Joint Genome Institute database [12], well-studied model organisms such as *E. coli* K12- W3110 and *Bacillus subtilis* strain 168 have ~86 and 81 % of their proteome functionally annotated, respectively [12]. However, the proteome of *Verrucomicrobium spinosum* DSM 4136 is only 48 % annotated. Ever more extreme than this is the feline parasite *Mycoplasma haemofelis *﻿, which has functional annotations for only 19 % of its proteome [12, 25]. With such a wide range of annotation coverage found among bacteria, we aimed to investigate the extent of annotation coverage across the bacterial tree of life, as well as to identify factors related to this important property of genomes.

## Methods

### Genome data sources

Bacterial genomes from AnnoTree [1] and their Pfam and KEGG (Kyoto Encyclopedia of Genes and Genomes) annotations (gtdb_r86_bac_genomic_files.tar.gz, gtdb_r86_bac_pfam_tophits.tar.gz and gtdb_r86_bac_ko_tophits.tar.gz, respectively) were accessed from https://data.ace.uq.edu.au/public/misc_downloads/annotree/r86/. Metadata for the downloaded genomes were retrieved from the Genome Taxonomy Database (GTDB) [2] at https://data.ace.uq.edu.au/public/gtdb/data/releases/release86/86.1/bac120_metadata_r86.1.tsv.

### Gene annotation

As described elsewhere, Pfam annotations were derived from Pfam v27.0 [9] and applied with hmmer v3.1b1 and Pfamscan (at ftp://ftp.ebi.ac.uk/pub/databases/Pfam/Tools/). KEGG [26] annotations were computed based on diamond v0.9.22 [27] matches against the UniRef100 dataset, members of which were pre-annotated with KEGG orthology (KO) annotations. The percentage of unannotated CDSs from the Pfam and KEGG approaches for each genome was calculated by comparing the number of CDSs in the metadata file with the number of CDSs with Pfam or KEGG matches in the Pfam and KO ‘tophits’ files from AnnoTree [1].

Genome annotation was also performed using Prokka v1.13.7 [11] with its default databases and with the rRNA and tRNA search options turned off. *Mycoplasmatales* (GTDB taxonomic nomenclature that includes *Entomoplasmatales* and *Mycoplasmatales* from the NCBI taxonomic nomenclature) was analysed with translation table 4, while GTDB orders *Absconditabacterales* and BD1-5 (which include candidate division SR1 and '*Candidatus* Gracilibacteria' from NCBI taxonomic nomenclature) were analysed with translation table 25. The unannotated class of CDSs were identified as those containing ‘hypothetical protein’ product names that also lacked Prokka database annotations. To analyse NCBI-derived protein annotations, we downloaded protein .gpff files associated with 113 424 genome IDs in the GTDB metadata file from NCBI’s ftp server (ftp://ftp.ncbi.nlm.nih.gov/genomes/all/). Any protein annotation in the ‘product’ line of the file containing the words ‘hypothetical’, ‘uncharacteri(s/z)ed protein’ or ‘unknown’ were counted towards the ‘unannotated’ fraction for that genome. The number of protein CDSs were also counted from the .gpff files for determining the percentage of unannotated CDSs. A data table containing the genome accession numbers and associated frequencies of annotated, unannotated and total gene counts produced by all three annotation pipelines is available online (https://github.com/doxeylab/genomeAnnotationCoverage).

### Statistical analyses

Statistical analyses were performed using R v3.2.3. For all statistical tests, the logarithm of genome size was used, which resulted in distributions closer to normality. The aov() function within the R base library was used to perform analysis of variance (ANOVA) tests and ANOVA [aov(),type=‘III’)] from the car v3.0–3 library was used to calculate analysis of covariance (ANCOVA) tests. Each ANCOVA identified a significant effect of the covariate GTDB taxonomic order on the annotation coverage, as well as a significant interference of the covariate with the effect of the independent variable. Linear regression was performed using the ggplot2 module stat_smooth(method=‘lm’).

The PubMed June 6 2019 database was downloaded using Entrez Direct. 'Research bias' represented by PubMed mentions was determined using Entrez Direct to search PubMed for all abstracts or titles that contained a genus name (NCBI taxonomic nomenclature).

Protein lengths were derived from the predicted proteins generated by Prokka [11].

## Results

### Annotation analysis

In order to explore patterns of genome annotation across bacteria, we analysed 27 372 bacterial genomes included as part of the AnnoTree database [1]. AnnoTree uses a phylogenetic tree originally derived from the GTDB [2] and allows users to visualize pre-computed functional annotations across the bacterial tree of life. We then examined three popular approaches for functional annotation that utilize different tools and databases, in addition to externally computed NCBI annotations, which we describe later. (i) Prokka [11] (v1.13.7): predicted proteins were annotated by blast+ searches against databases of curated proteins, and by hmmscan [28] searches against the hamap HMMs library [29]. (ii) KEGG [26]: predicted proteins were annotated with KO numbers based on diamond [27] searches against the KEGG database. (iii) Pfam [9]: predicted proteins were annotated by hmmscan searches against the Pfam-A HMM library.

Following annotation with these pipelines, for every genome, we then subdivided predicted CDSs into two categories: (i) *annotated proteins* – sequences matched to either functionally characterized or unnamed families; and (ii) *unannotated proteins* – sequences without any matches. CDSs matching protein families without an annotated molecular function were still included in the first group, since these domains may still possess limited information that can be transferred to a new sequence.

**Fig. 1.**
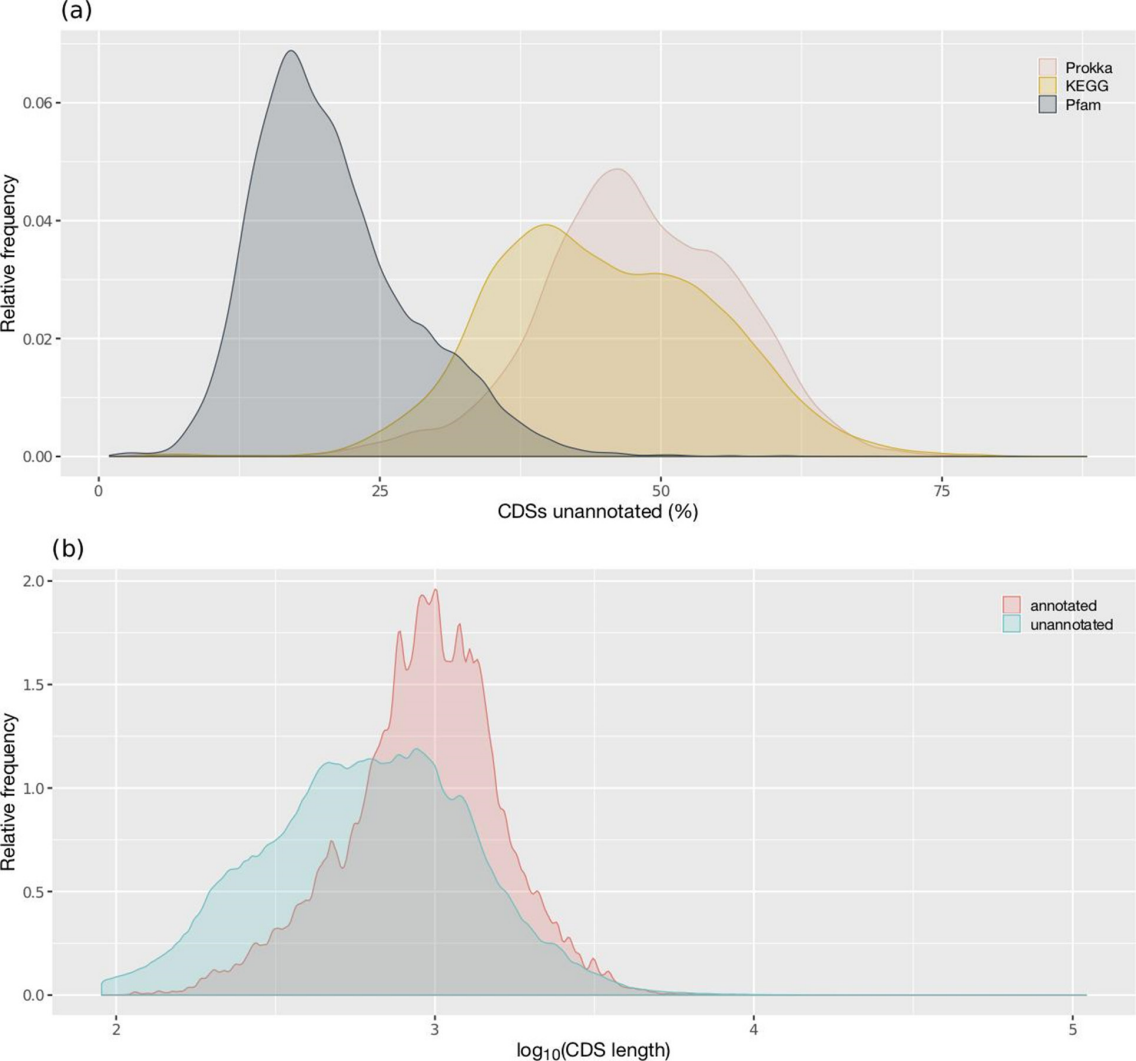
Distributions of genome annotation incompleteness across GTDB bacteria and length of annotated versus unannotated CDSs. (a) Relative frequency distribution of annotation coverage based on annotation with Prokka, KEGG and Pfam. (b) Relative frequency distribution of the length (bp) of CDSs in genomes present in AnnoTree. Annotation status was determined with our binary Prokka classification. The lowest length for both annotated and unannotated sequences is 90 bp, due to the length threshold in Prodigal [32].

Based on Prokka results, the mean proteome annotation coverage was 52 ±9 % (48 % unannotated) (Fig. 1a). This is expectedly lower than that reported for model organisms and higher than that reported for the low-end cases described earlier. It is worth noting that the default Prokka parameters for functional annotation are fairly strict, as only reference proteins with experimental evidence are considered for functional assignments [11], and that annotation coverage can potentially be increased by adding custom databases of curated annotations. The KEGG-based annotation method produced similar results with 55±10 % mean annotation coverage (Fig. 1a). The third approach based on Pfam domain-based annotation produced a mean of 79±7.1 % annotation coverage (Fig. 1a), which is higher than that of the other methods. To compare our results against externally derived functional annotations, we also examined 113 424 previously annotated proteomes within the NCBI database. We calculated a mean annotation coverage of 79.8±10 % for these proteomes (see Methods).

We observed a trend for unannotated protein sequences to be shorter in length (Fig. 1b). Shorter proteins can be more difficult to annotate due to poor database coverage, lower match scores and an increased chance of being pseudogenes (one signature of pseudogenization is the accumulation of premature stop codons, which leads to shorter CDSs) [30]. While it is challenging to uncover pseudogenes at such a large scale [31, 32], there was an observable difference in the length distribution of the unannotated sequences, consistent with an increased proportion of pseudogenes. Despite this, a large proportion of the distribution was indistinguishable from that of annotated sequences (Fig. 1b).

With all annotation pipelines analysed, we observed extreme variation in annotation incompleteness across bacterial genomes (Fig. 1a). For example, based on protein homology searching using Prokka, annotation incompleteness ranged from 2.3 % ('*Candidatus* Baumannia cicadellinicola') to 85.5 % (* Mycoplasma haemofelis* Ohio2). Similar values were obtained using KEGG-based annotation, with incompleteness ranging from 3.1 % ('*Candidatus* Evansia muelleri') to 87.9 % (* Algoriphagus boritolerans *). Next, to further explore factors influencing this variation, we explored the relationship between annotation coverage and various features, such as taxonomy, genome size and research bias.

### Taxonomy

**Fig. 2.**
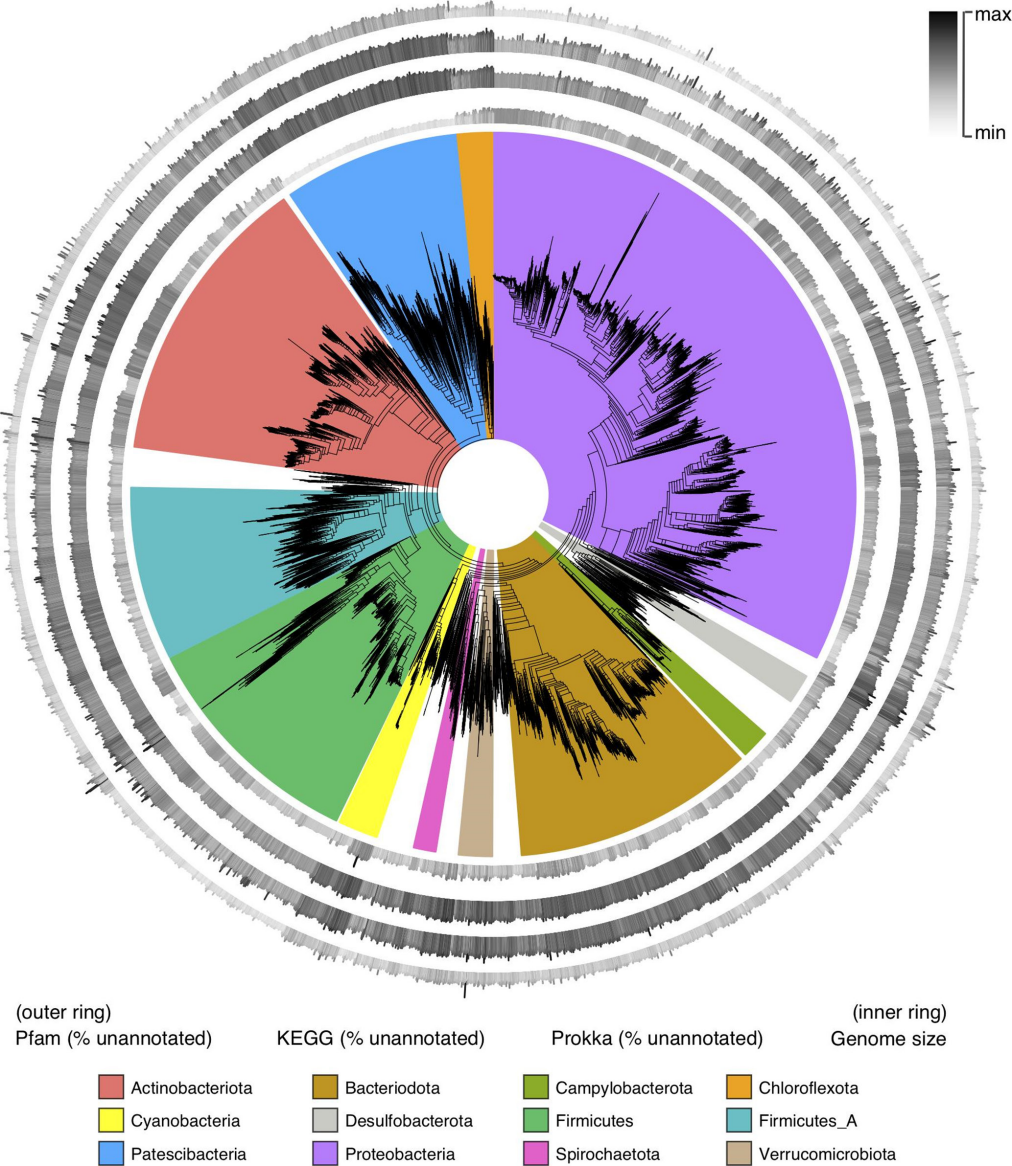
Genome annotation incompleteness across the bacterial tree of life. Annotation incompleteness has been mapped to the outer edges of the tree of life obtained from AnnoTree [1], which was originally derived from the GTDB [2]. The height of each bar (and colour) depicts traits (annotation incompleteness and genome size), which have been normalized separately for each metric. For annotation incompleteness, the gradient goes from 0 % (minimum) to 100 % (maximum). Four metrics are shown, including annotation incompleteness as determined using Prokka (outer ring), followed by that determined using Pfam, that determined using KEGG and genome size (inner ring).

**Fig. 3.**
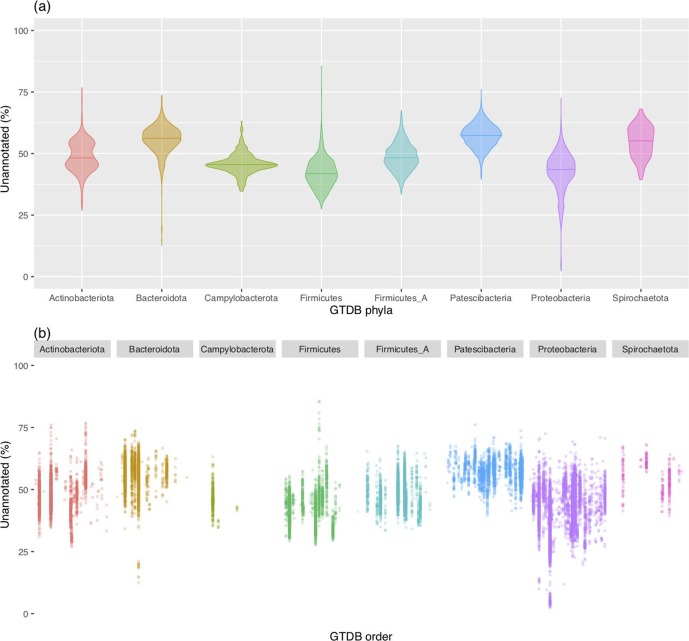
Distributions of genome annotation coverage subdivided by taxonomic group. Genomes were annotated using Prokka with default parameters (see Methods). Only the most common phyla from the GTDB [2] are shown. (a) Taxonomic separation by phyla. (b) Taxonomic separation by order. Labelled orders are using GTDB taxonomic nomenclature.

To study the potential taxonomic bias in genome annotations, we mapped annotation completeness onto the bacterial phylogeny, and partitioned it according to the taxonomic scheme defined by the GTDB (Fig. 2). Differences in annotation coverage were visually apparent across the tree, and a strong degree of clade-specific patterns could be observed. This taxonomic annotation bias was supported by quantitative measurements at different taxonomic levels (Fig. 3). Even at the phylum level, we observed differences in genome annotation coverage between taxa (Fig. 3a; ANOVA *P* value <2×10^−16^), with greater resolution revealed at every subsequent taxonomic level (Fig. 3b). This taxonomic effect was consistent between Prokka (Fig. 3a, b), KEGG (Fig. S1a, available with the online version of this article; ANOVA *P* value <2×10^−16^) and Pfam (Fig. S1b; ANOVA *P* value <2×10^−16^) proteome annotations. *Patescibacteria*, a phylum recently formed from the highly underrepresented candidate phyla radiation associated with smaller genomes [33, 34], had the highest mean of unannotated CDSs across all three annotation systems. *Spirochaetota *, a smaller phylum, and *Bacteroidota *, found across many environments, also had higher unannotated proportions (54.8 % mean and 55.7 % mean, respectively). *Proteobacteria* and *Firmicutes *, the phyla of the majority of bacterial model organisms, had better annotation completeness across all three annotation systems with mean unannotated proportions of 42.6 and 42.3 %, respectively. Thus, the taxonomic bias on genome annotation completeness may be in part due to what can be described as research bias or model organism bias (a larger scientific community effort towards functional characterization), which we explore further in a later section.

### Genome size

**Fig. 4.**
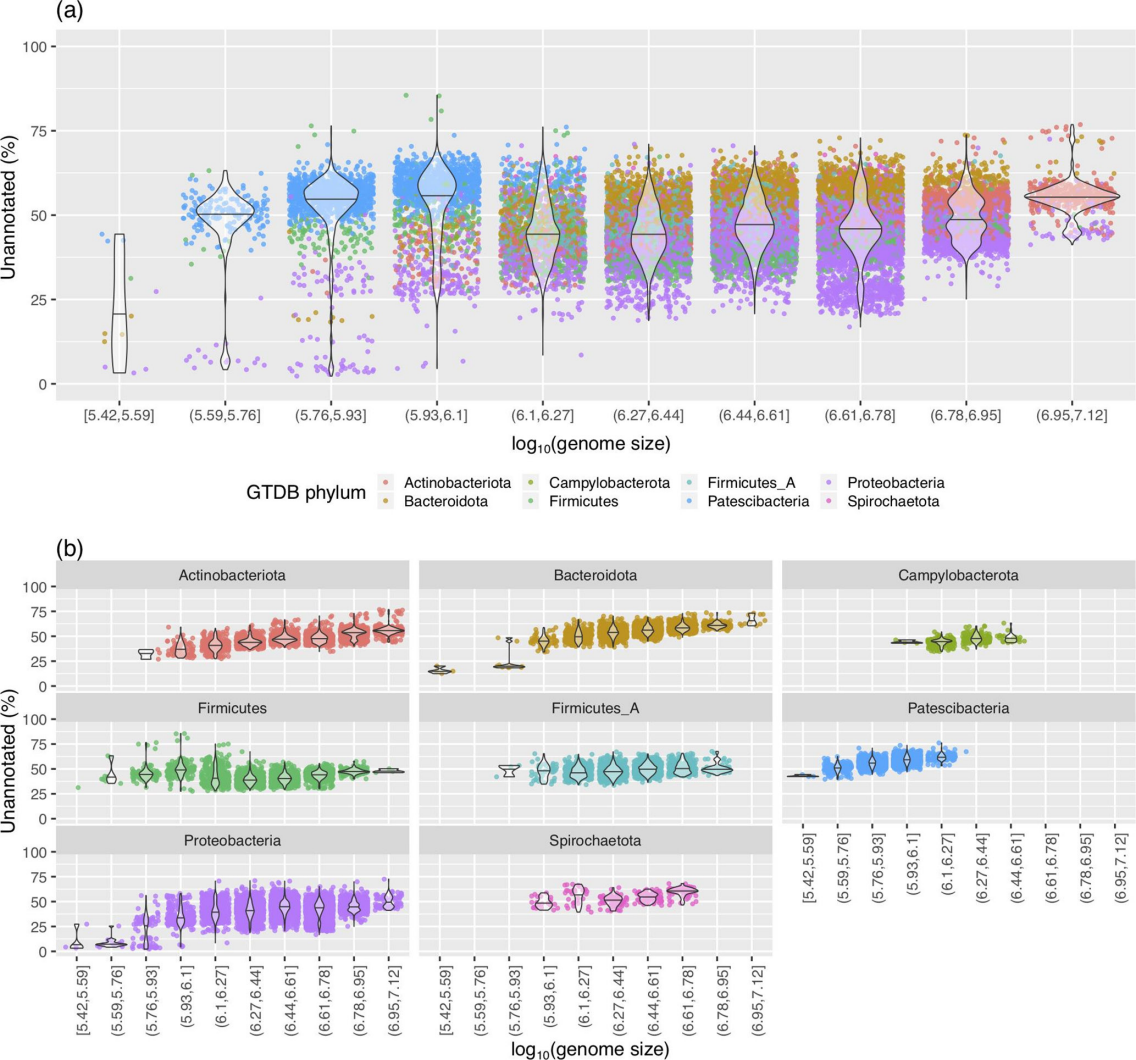
Effect of genome size (bp) on Prokka genome annotation coverage. The log_10_(genome size in bp) is binned into 10 distinct bins to better display the trend. Square and open brackets indicate intervals that include and do not include the adjacent number, respectively. (a) Only the most common GTDB phyla are shown. (b) The most common GTDB phyla are displayed separately.

Genome size, a trait related to taxonomy (as evident in Fig. 2), also appeared to affect the annotation coverage of genomes. Even without accounting for the confounding impact of taxonomy, a clear relationship between genome size and genome annotation completeness was visible (Fig. 4a). A closer look at this phenomenon within individual phyla revealed an even clearer picture of this trend, where larger genomes were associated with a larger proportion of unannotated proteins [Fig. 4b, S2a (KEGG) and S2b (Pfam)].

**Fig. 5.**
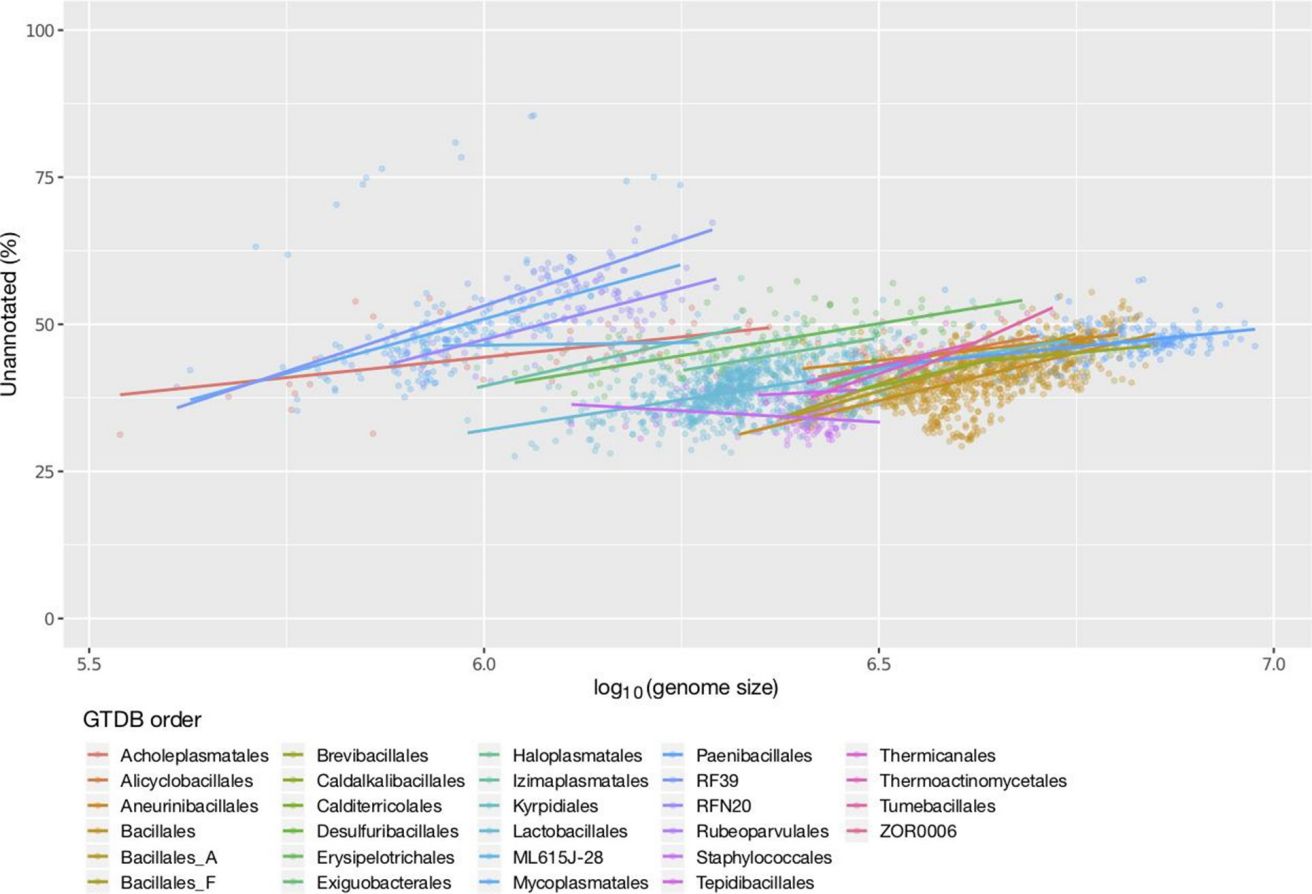
Prokka genome annotation coverage of *Firmicutes* (GTDB taxonomy) against genome size. Trend lines are displayed for each taxonomic order.

An interesting case demonstrating this relationship is the phylum *Firmicutes *. Although at a phylum level, the effect of genome size on annotation completeness was not entirely clear (Fig. 4), when subdivided into lower taxonomic levels (Fig. 5), the trend was readily apparent. That is, different taxonomic groups within the *Firmicutes* possessed distinct distributions of genome completeness and each was also influenced by genome size. For example, *Mycoplasmatales *, RF39 and RFN20 (GTDB taxonomic nomenclature [2]) possess relatively small genomes, but had a high fraction of unannotated CDSs. Yet, within these taxonomic groups, genome size positively correlated with the level of annotation incompleteness. Thus, these cases illustrate how annotation incompleteness is driven by multiple factors.

Consistent with these observations, an ANCOVA test controlling for the GTDB taxonomic order revealed a significant relationship between genome size and annotation incompleteness for Prokka, KEGG and Pfam annotations (*P* value=3.6×10^−5^, 2.5×10^−3^ and 1.1×10^−4^, respectively). The protein annotations in the NCBI database also showed a significant difference between taxonomic phyla (ANOVA *P* value <2.2×10^−16^; Fig. S3a) and a relationship with genome size (ANCOVA, while controlling for GTDB taxonomic orders, *P* value=2.3×10^−10^; Fig. S3b). Since the largest factor influencing genome size variation in bacteria is the gain and loss of 'accessory' genes [35, 36], it can be reasoned that this trend may reflect an increased difficulty in functional annotation of accessory genes versus 'core' genes (see Discussion). Since genome size is also related to other factors such as G+C content, we also examined the correlation between G+C content and annotation completeness. However, this relationship was not as clear (Fig. S4) and was non-significant when controlling for taxonomy (ANCOVA *P* values of 0.6, 0.85 and 0.33 for Prokka, KEGG and Pfam, respectively).

### Research bias

To explore the effects of research bias on annotation coverage, we counted the number of times each genus was mentioned in abstracts or titles within the PubMed database, and also examined genome publication date. Here, we adopted NCBI taxonomic nomenclature as it has been used more frequently. Genera with over 75 000 mentions (such as *Escherichia *, *Staphylococcus* and *Pseudomonas *) generally had a greater annotation coverage compared to genera that occurred less frequently in publications [Figs S5a (Prokka), S5b (KEGG), S5c (Pfam)]. Similarly, genomes released before 2003 tended to have a greater proportion of annotated CDSs [Figs S6a (Prokka), S6b (KEGG), S6c (Pfam)]. However, these effects were only apparent in the extreme cases (i.e. model organisms associated with extreme publication volume). Moreover, the majority of genera in this uppermost bracket were *Proteobacteria* and *Firmicutes *, consistent with our earlier analysis of taxonomic influence on genome annotation coverage.

**Fig. 6.**
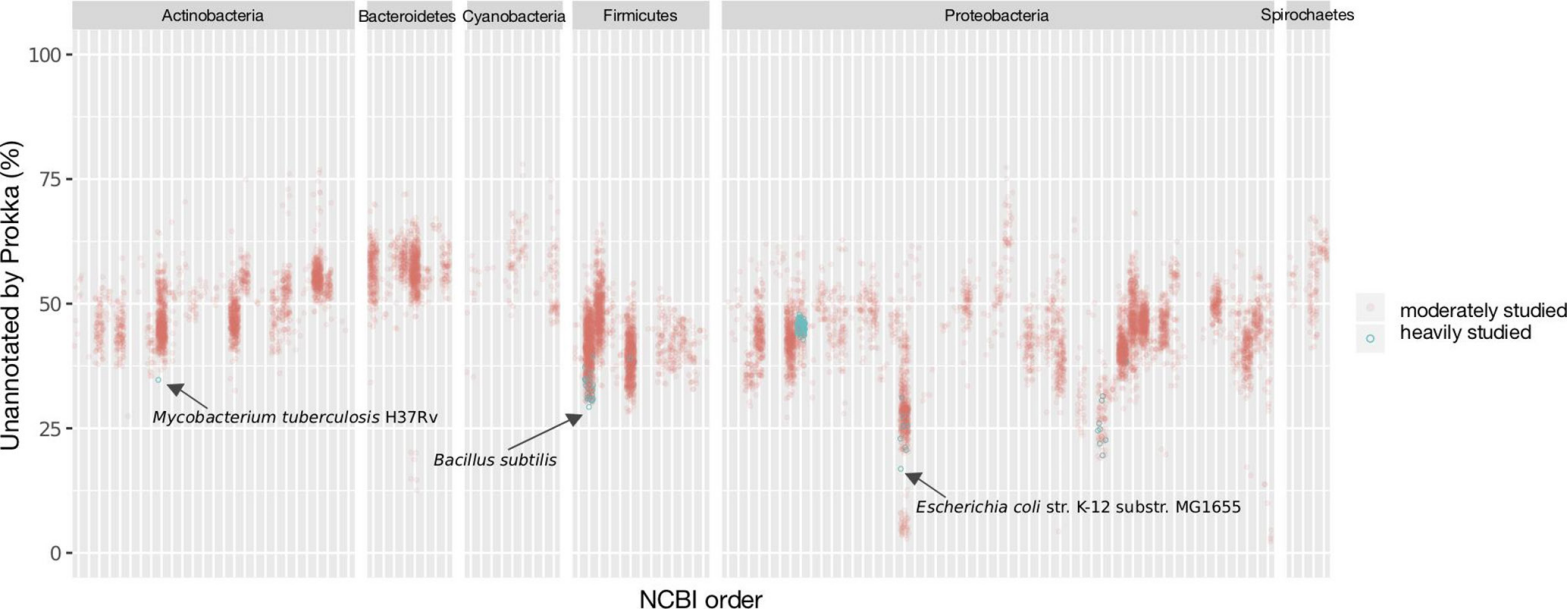
Influence of research bias on genome incompleteness. The top six most abundant phyla are shown and each is further subdivided by taxonomic order. Orders appear as distinct vertical columns. Heavily studied genomes, as measured by PubMed abstract counts per species (>15 000), show a marked reduction in unannotated sequences (annotated with Prokka) compared to other moderately studied genomes (500–1000) in their taxonomic group. Other heavily studied species include *Listeria monocytogenes *, *Staphylococcus aureus *, *Streptococcus pneumoniae *, *Helicobacter pylori *, *Klebsiella pneumoniae *, *Haemophilus influenzae* and *Pseudomonas aeruginosa *. It must be noted that the terms ‘heavily’ and ‘moderately’ studied organisms are relative, are associated only with the frequency of published papers, and do not account for the true impact of publications and other work that contribute toward functional annotation.

To explore this phenomenon further, we examined the distributions of genome annotation completeness while subdividing by taxonomy, mapping only the most heavily studied taxa onto their respective lineages. This clarified the effect of research bias since model organisms (e.g. *E. coli *, *Bacillus subtilis *, *Mycobacterium tuberculosis *) stood out as being among the best annotated genomes in their respective taxonomic groups (Fig. 6). There were, however, some exceptions to this phenomenon; within the *Proteobacteria *, a noticeable group of organisms had annotation completeness well exceeding that of *E. coli *. These organisms included endosymbionts with highly reduced genomes, such as *Buchnera aphidicola *, an endosymbiont of aphids, '*Candidatus* Blochmannia' (an ant symbiont), *Wigglesworthia* (a symbiont of tsetse flies) and others. This may be due to multiple factors, including an increased proportion of core or 'essential' functions associated with ‘minimal genomes’ and, thus, easier-to-annotate processes in reduced genomes of parasitic organisms [37–39], as well as the close evolutionary relationship of these genomes to the heavily studied model organism *E. coli* [40, 41].

## Discussion

As genomes shape our understanding of organism function, not only individually but also as a community, it is important to assess our ability to annotate genomes across the tree of life and understand the factors that influence this important property. Here, we used the GTDB [2] and AnnoTree [1] in combination with various annotation pipelines to perform a comprehensive assessment of genome annotation coverage across the bacterial phylogeny. Our analysis revealed extreme variation in genome annotation coverage across and within taxonomic groups. Numerous factors appear to influence levels of annotation completeness across bacterial genomes, including annotation method, taxonomy, genome size and research bias.

Overall, the mean annotation completeness of bacterial genomes varied from ~52 % for methods requiring high-stringency matches to reference proteins, to 79 % for more sensitive domain-based annotation methods. While domain-based annotation methods produced the highest proportion of annotated CDSs, these estimates of annotation coverage may be not be realistic, since the mere presence of a domain in a predicted protein sequence is not necessarily sufficient to assign function, and consideration of domain architecture is more informative. Also, although three annotation pipelines were performed separately, a combination of methods would have likely resulted in greater annotation coverage, as observed in previous studies [6]. However, the goal of this study was not to optimize annotation coverage across bacteria, but rather to assess it using standard, commonly used pipelines.

Taxonomy was an important factor influencing genome annotation completeness. Some of this taxonomic bias may stem from research bias, whereby genomes that are more closely related to those of model organisms possess a greater chance of being successfully annotated based on detectable homology. Indeed, phyla containing many model organisms were found to have, on average, more annotated CDSs than their understudied counterparts. In addition, within broader taxonomic groups, specific model organisms (e.g. *E. coli *) stood out as outliers in terms of annotation coverage. This pattern was also demonstrated for other highly studied species as determined based on publication volume (occurrences of species names in PubMed abstracts and titles).

Our analysis also uncovered an interesting, significant anticorrelation between genome size and annotation coverage, which was consistently detected across a range of taxonomic groups. Larger genomes showed lower annotation coverage, which suggests a relative lack of annotations and functional characterization concerning accessory proteomes. One interpretation of this finding is that core proteomes contain more essential and widely studied processes, resulting in increased genome annotation coverage. In contrast, the accessory gene content within a pangenome of a species may include a more diverse repertoire of genes, including those derived from prophages [35] and integrated elements, which are known to be particularly challenging for annotation [42]. The dynamic accessory genome of a species may also possess increased pseudogene content, resulting in shorter (truncated) and potentially divergent ORFs that are harder to assign function through homology searches. The observed difference in the length distribution of annotated versus unannotated CDSs is consistent with this idea.

The reduced genomes of symbionts and parasites are extreme examples of how factors related to genome size may affect annotation completeness. In our analysis, reduced genomes were found at both ends of the spectrum of annotation completeness. Within the *Firmicutes *, for example, some parasitic genomes in the *Mycoplasmatales* were poorly annotated. This may be a result of increased pseudogene content, which is thought to accumulate in the reduced genomes of some organisms due to genetic drift [35, 43–45]. However, the reduced genomes of endosymbiotic *Proteobacteria* such as *Buchnera aphidicola* were extremely well annotated, consistent with previous analyses [46], which may be due to efficient purging of genes and pseudogenes over a longer evolutionary timescale with retention of core processes. These core or essential functions are in turn easier to annotate bioinformatically [for previous papers on the minimal genome concept see references by Mushegian (1999) and Koonin (2000) [38, 39]]. Their increased annotation completeness may also in part benefit from their close relationship with a model organism (* E. coli *).

Finally, our analysis highlighted certain lineages (e.g. the *Patescibacteria* within the candidate phyla radiation group) as possessing a higher level of hypothetical gene content. This may reflect the presence of highly divergent gene families that escape the detection limits of standard homology-based annotation, or this may be indicative of new protein functions, metabolic activities and biological traits. To assign function to these sequences, the use of powerful/sensitive methods for protein function prediction may be useful; these include remote-homology detection and structure prediction approaches [18, 47]. Methods for function prediction will also benefit from continual expansion of Gene Ontology and other controlled vocabularies [48, 49]. In addition to sequence-to-function methods, a complementary ‘function-to-sequence’ type of approach may also be useful, where a required parts list of functions is used to guide the search for potential gene functions [50]. Finally, our ability to assign function computationally to these and other bacterial genomes is inherently tied to the quantity and quality of experimentally derived functional information contained within references databases. Continued experimental characterization of understudied organisms and hypothetical/novel gene families will be critical to widen the net of annotation coverage and lead to more accurate genome analyses and functional insights derived from genomic and metagenomic studies.

## Data Bibliography

1. Lobb B. Genome accession numbers and frequencies of annotated versus unannotated genes produced by three annotation pipelines: https://github.com/doxeylab/genomeAnnotationCoverage (2019).

## Supplementary Data

Supplementary material 1
