# Supplementary material for: An assessment of genome annotation coverage across the bacterial tree of life
Source: Microb Genom. 2020 Mar 3;6(3):e000341. doi: 10.1099/mgen.0.000341 (PMC7200070; doi:10.1099/mgen.0.000341)
Supplement: Supplementary material 1 [file mgen-6-341-s001.pdf]

Supplementary Information

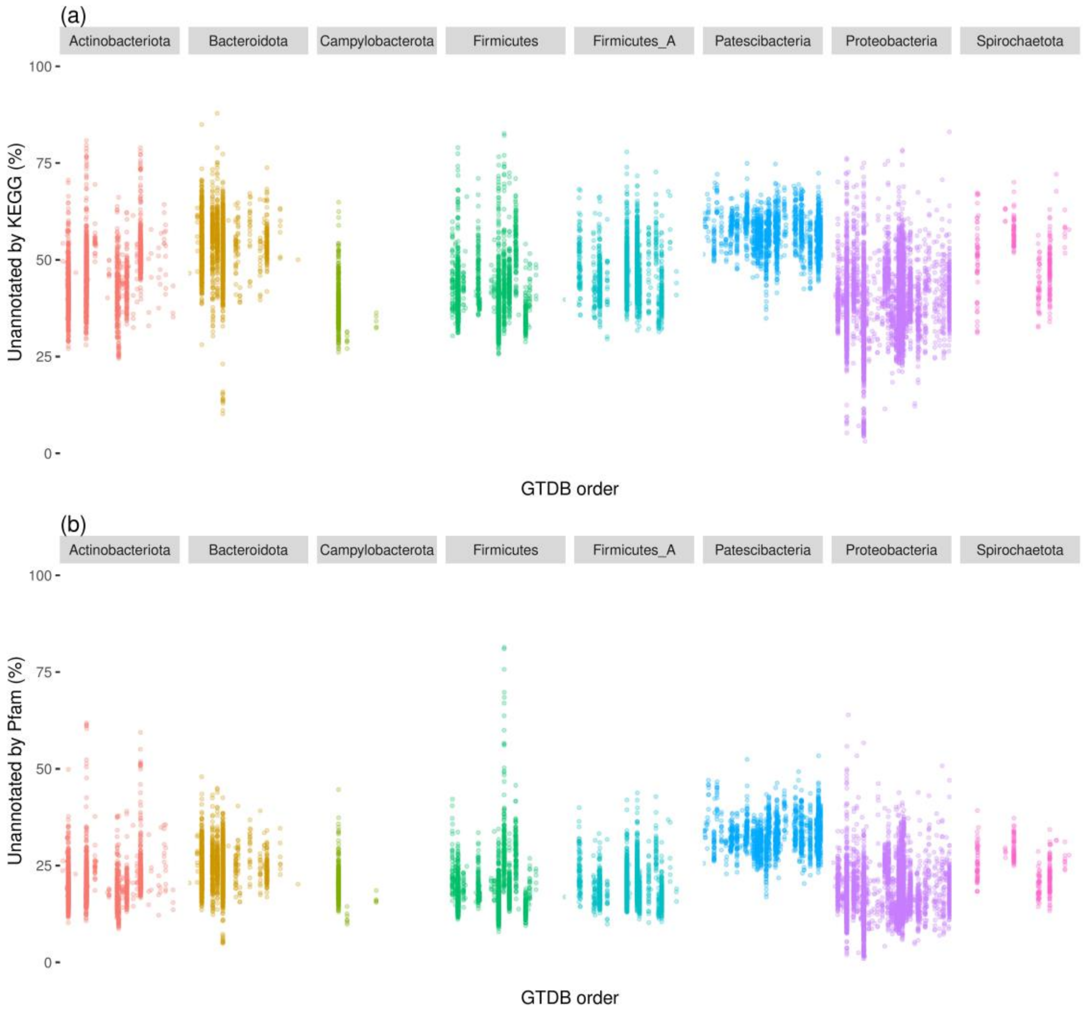

Fig. S1. Taxonomic separation of genome annotation coverage by order using the taxonomic nomenclature from the Genome Taxonomy Database (GTDB). Only the most common GTDB phyla are shown. (a) KEGG genome annotation. (b) Pfam genome annotation.

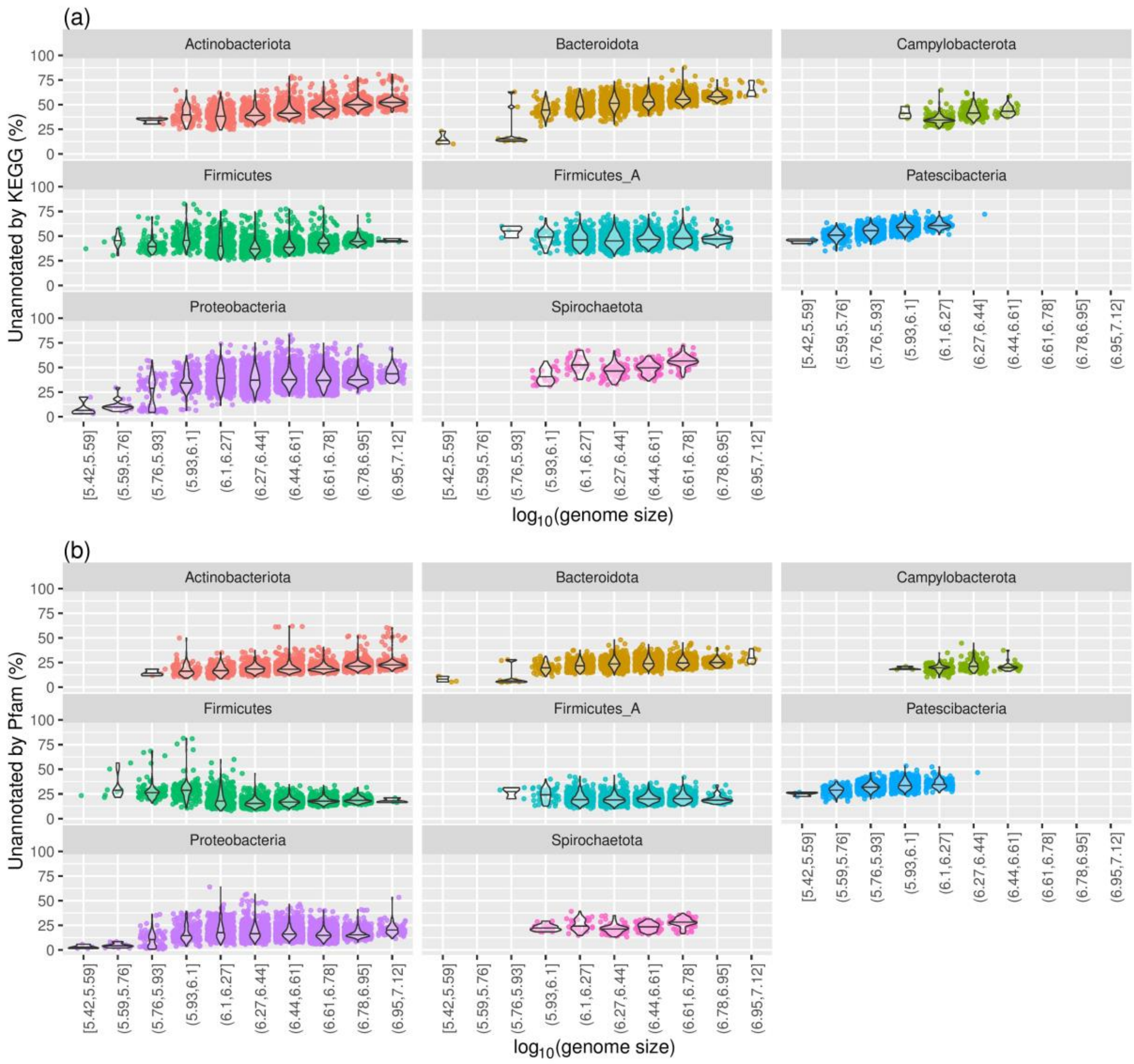

Fig. S2. Effect of genome size (bp) on genome annotation coverage. $\log_{10}$(genome size) is binned into 10 distinct bins to better display the trend. The most common GTDB phyla are displayed separately. (a) KEGG genome annotation. (b) Pfam genome annotation.

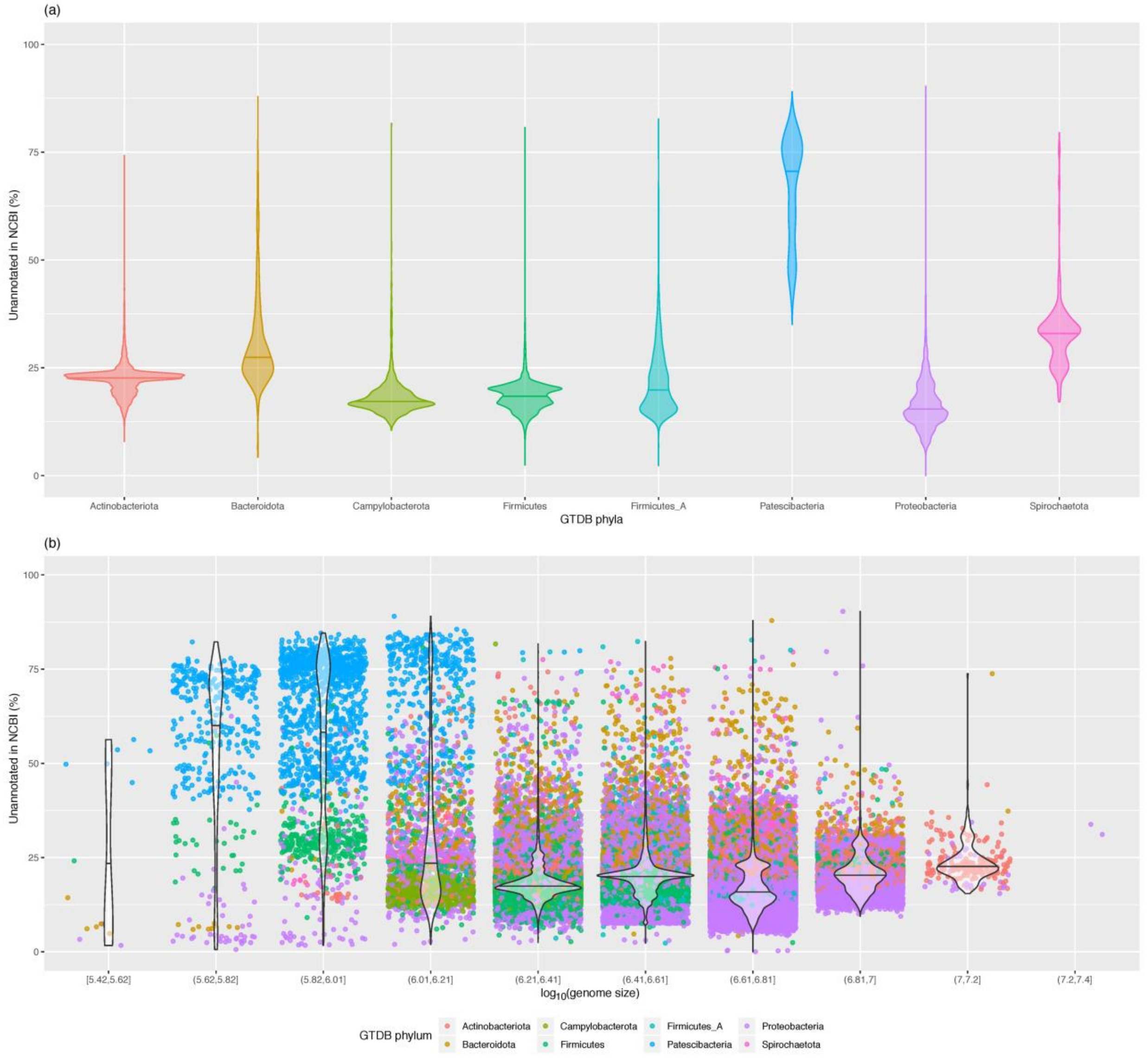

Fig. S3. Genome annotations from NCBI (a) Taxonomic separation of genome annotation coverage by phyla using the taxonomic nomenclature from the Genome Taxonomy Database (GTDB). Only the most common GTDB phyla are shown. (b) Effect of genome size on genome annotation coverage in NCBI. $\log_{10}$(genome size) is binned into 10 distinct bins to better display the trend. The most common GTDB phyla are displayed.

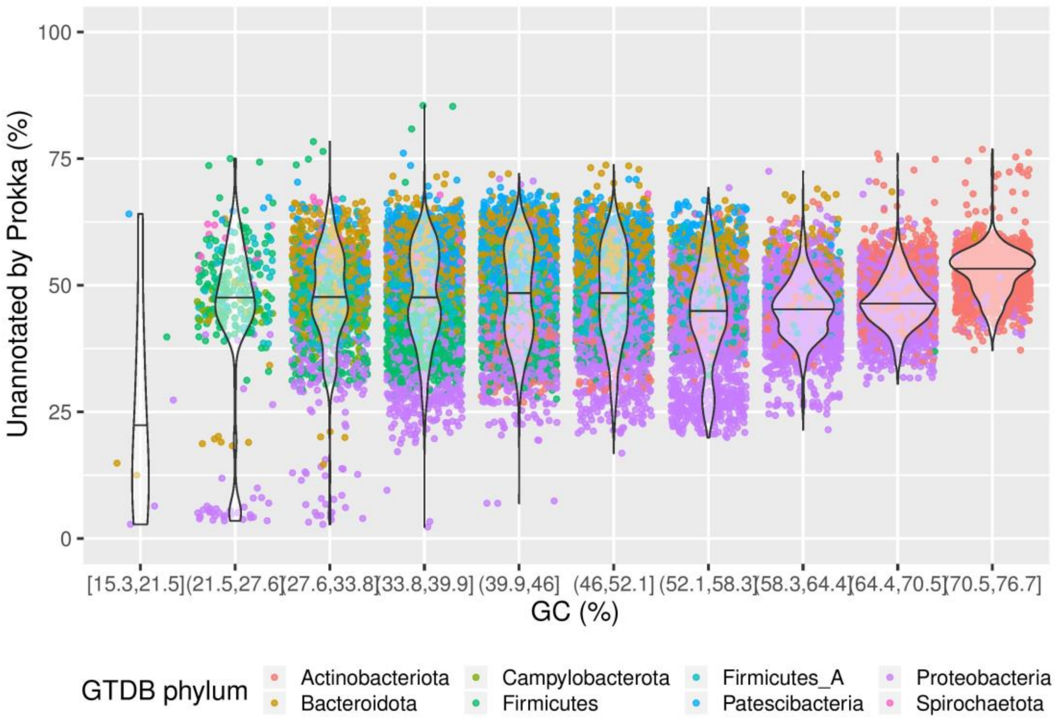

Fig. S4. Effect of GC percentage on genome annotation coverage by Prokka. Percent GC is binned into 10 distinct bins to better display the trend. The most common GTDB phyla are displayed.

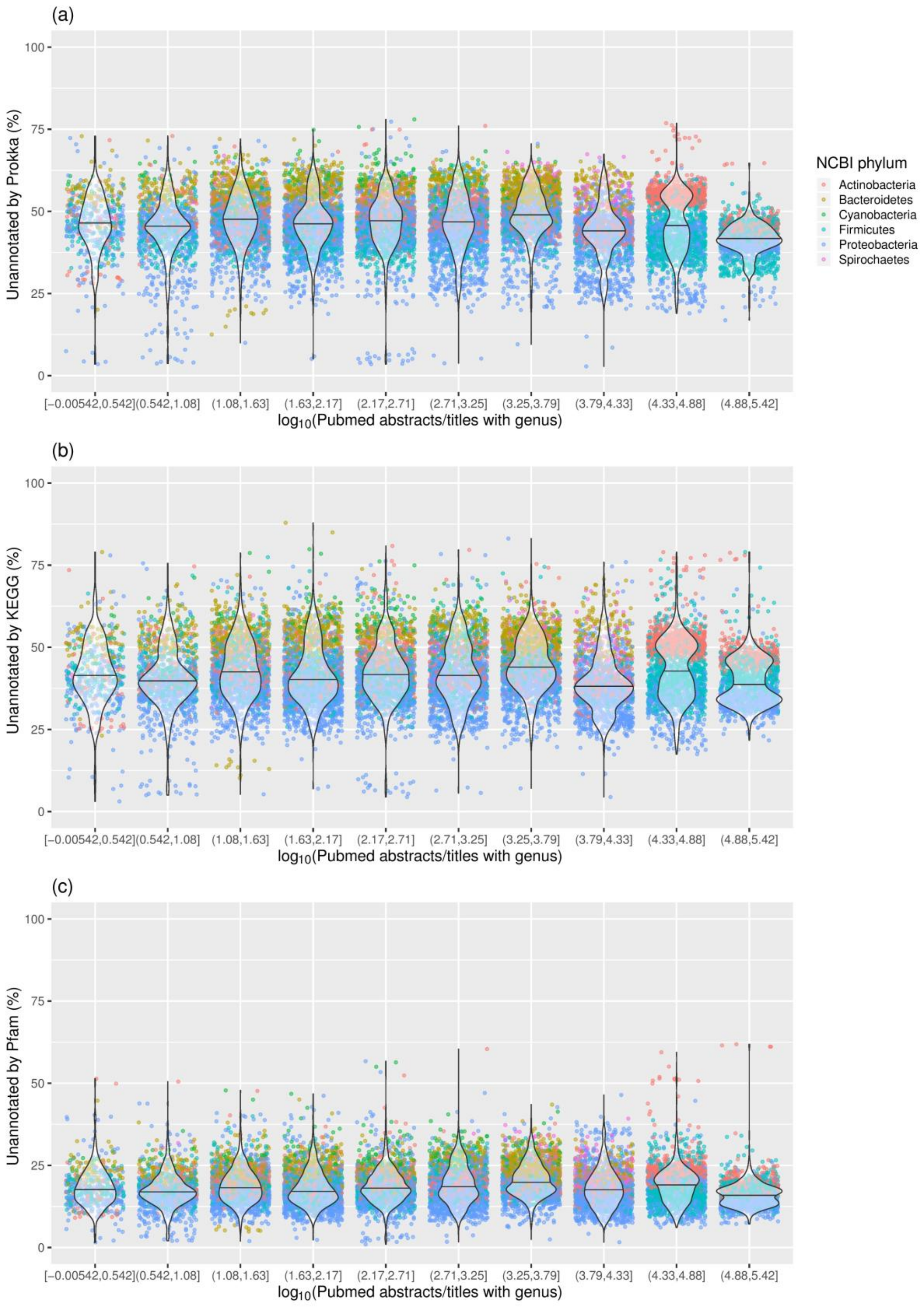

Fig. S5. $\log_{10}$(Pubmed mentions of genera in titles or abstracts) by genome annotation coverage. The Pubmed mentions are binned into 10 distinct bins to better display the lack of any significant

trend. Only the most common phyla according to NCBI taxonomic nomenclature are shown. (a) Prokka genome annotation; (b) KEGG genome annotation; (c) Pfam genome annotation.

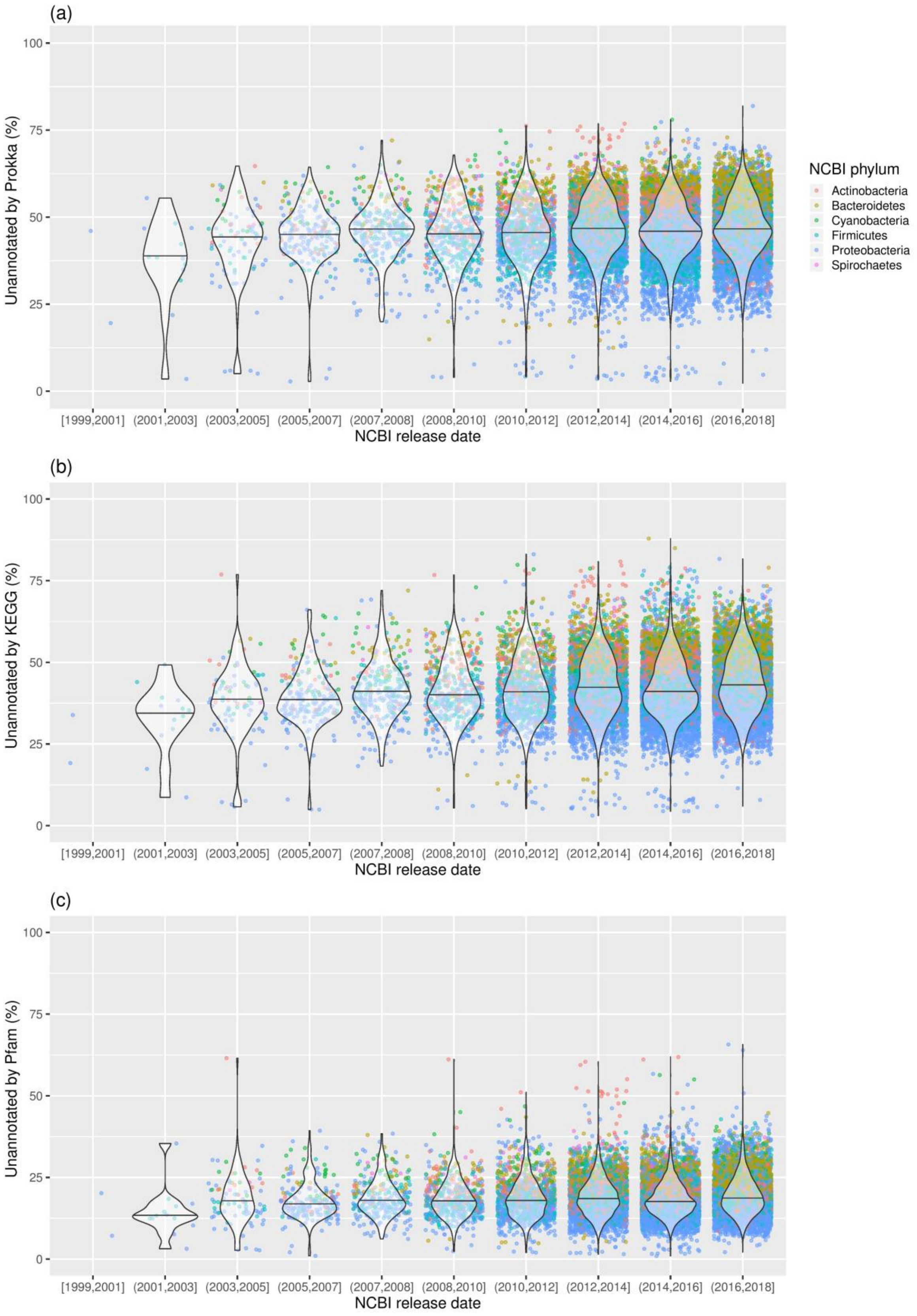

(a)
Unannotated by Prokka (%)
NCBI release date
NCBI phylum
Actinobacteria
Bacteroidetes
Cyanobacteria
Firmicutes
Proteobacteria
Spirochaetes
(b)
Unannotated by KEGG (%)
NCBI release date
(c)
Unannotated by Pfam (%)
NCBI release date
[1999,2001]
(2001,2003]
(2003,2005]
(2005,2007]
(2007,2008]
(2008,2010]
(2010,2012]
(2012,2014]
(2014,2016]
(2016,2018]
0
25
50
75
100

Fig. S6. NCBI genome release date by genome annotation coverage. The NCBI release date has been binned into 10 distinct bins to better display the lack of any significant trend. Only the most common phyla according to NCBI taxonomic nomenclature are shown. (a) Prokka genome annotation; (b) KEGG genome annotation. (c) Pfam genome annotation.
